# The efficacy and safety of intraoperative intravenous amiodarone in patients undergoing on-pump coronary artery bypass grafting surgery: a systemic review and PRISMA-compliant meta-analysis

**DOI:** 10.1186/s13019-024-02732-9

**Published:** 2024-05-03

**Authors:** Jin-He Deng, Bin Jia, Yun-Tai Yao, The Evidence in Cardiovascular Anesthesia (EICA) Group

**Affiliations:** 1 Department of Anesthesiology, Guangdong Provincial Hospital of Chinese Medicine, (The Second Affiliated Hospital of Guangzhou University of Chinese Medicine), Guangdong Province, Guangzhou, 510000 China; 2 Department of Anaesthesiology, Fuwai Hospital, National Center for Cardiovascular Diseases, Peking Union Medical College and Chinese Academy of Medical Sciences, No. 167, Beilishi Road, Xicheng District, Beijing, 100037 China

**Keywords:** Amiodarone, Atrial fibrillation, Coronary artery bypass grafting, Cardiopulmonary bypass

## Abstract

**Background:**

To evaluate the clinical efficacy and safety of intraoperative intravenous amiodarone for arrhythmia prevention in on-pump coronary artery bypass grafting (CABG) patients.

**Methods:**

A meta-analysis of randomized controlled trials was conducted. Pubmed, Embase, Cochrane Library, Ovid, China National Knowledge Infrastructure, and the Wan Fang database until July 1th, 2023. The primary outcomes of interest included the incidences of intra- and post-operative atrial fibrillation (POAF), ventricular fibrillation, or any arrhythmia, including atrial fibrillation, ventricular fibrillation, ventricular tachycardia, premature ventricular contraction, and sinus bradycardia. For continuous and dichotomous variables, treatment effects were calculated as the weighted mean difference (WMD)/risk ratio (RR) and 95% confidence interval (CI).

**Results:**

A database search yielded 7 randomized controlled trials including 608 patients, where three studies, including three treatments (amiodarone, lidocaine, and saline), contributed to the clinical outcome of atrial fibrillation, ventricular fibrillation, or any arrhythmia. Meta-analysis demonstrated that amiodarone can significantly reduce the incidence of POAF (RR, 0.39; 95%CI: 0.20, 0.77; *P* = 0.007, *I*^*2*^ = 0%) in patients undergoing on-pump CABG; there was no statistically significant influence on intra-operative atrial fibrillation, intra- and post-operative ventricular fibrillation, or any arrhythmia.

**Conclusions:**

The current study suggests that intraoperative administration of intravenous amiodarone may be safe and effective in preventing POAF in patients undergoing on-pump CABG. More well-designed clinical trials are needed to validate this result.

**Supplementary Information:**

The online version contains supplementary material available at 10.1186/s13019-024-02732-9.

## Introduction

Reperfusion arrhythmia is one of the common complications after on-pump coronary artery bypass grafting (CABG). Supraventricular (especially atrial fibrillation) and ventricular arrhythmia are among the most commonly encountered postoperative complications associated with CABG [1]. Studies have estimated the incidence of postoperative atrial fibrillation (POAF) to be as high as 10%-30% following cardiac surgery [2] or 40%-60% after CABG or cardiac valve surgery [3, 4]. The reported prevalence of ventricular fibrillation after aortic cross-clamping release (ACCR) in patients undergoing CABG ranged from 70 to 90% [5, 6]. Perioperative arrhythmia is associated with a longer length of stay in the hospital and increased rates of morbidity and mortality in cardiac surgical patients [5–8]. The current clinical practice guideline recommends the use of beta-blocker therapy as prophylaxis for tachyarrhythmia in all patients undergoing cardiac surgery [7]. However, there are many cases where beta-blocker therapy is contraindicated; thereby, the use of amiodarone prophylaxis for tachyarrhythmia is recommended in these situations [9].

Amiodarone is a class III anti-arrhythmic agent mainly used for ventricular and supraventricular arrhythmias [10–12]. Evidence has suggested that perioperative oral amiodarone could effectively reduce the incidence of POAF in patients undergoing CABG [13]. Amiodarone has emerged as the leading antiarrhythmic therapy for the termination and prevention of ventricular arrhythmia in different clinical settings because of its proven efficacy and safety. In patients with shock-refractory out-of-hospital cardiac arrest and hemodynamically destabilizing ventricular arrhythmia, amiodarone is the most effective drug available to assist in resuscitation [14]. However, little is known about the use or effectiveness of intraoperative amiodarone.

As the evidence supporting the routine intraoperative use of amiodarone to prevent arrhythmia in patients undergoing on-pump CABG remains weak, we performed a systemic review and meta-analysis to evaluate the clinical efficacy and safety of intraoperative intravenous amiodarone for arrhythmia prevention in on-pump CABG patients.

## Methods

We conducted a systemic review and meta-analysis according to the Preferred Reporting Items for Systemic Reviews and Meta-Analysis Quality of Reporting of Meta-Analysis (PRISMA) Guidelines [15]. The protocol of the current meta-analysis has been registered on the International Prospective Systematic Reviews Registry database (PROSPERO: CRD42022377134).

### Search strategy

Potential relevant randomized controlled trials (RCTs) were searched from Pubmed, Embase, Cochrane Library, Ovid, China National Knowledge Infrastructure (CNKI), and the Wan Fang database until July 1th, 2023, by using different combinations of search words (titles, key words, or mesh terms) as follows: (cardiac surgical or cardiopulmonary bypass, or coronary artery bypass; amiodarone and randomized controlled trial, controlled clinical trial, randomized or randomly (Supplement Table 1). An English language restriction was used. Additionally, we used the references from the retrieved articles to further identify relevant studies.

### Inclusion criteria and exclusion criteria

We included all RCTs comparing the efficacy and safety of intraoperative intravenous injection of amiodarone with controls (lidocaine or saline) on adults undergoing on-pump CABG. Primary outcomes of interest included the incidences of intra-, and post-operative AF, ventricular fibrillation, or any arrhythmia, including atrial fibrillation (AF), ventricular fibrillation (VF), ventricular tachycardia (VT), premature ventricular contraction (PVC), and sinus bradycardia (SB). Secondary outcomes of interest included defibrillation incidence, defibrillation energy, inotropic requirement, mechanical ventilation duration (MVD), length of stay (LOS) in the intensive care unit (ICU), LOS in the hospital, heart rate (HR), mean arterial pressure (MAP), and Pondus hydrogenii (PH). Exclusion criteria included studies published as review articles, case reports or abstracts; studies based on animal models; duplicate publications; non-English language literature; and studies lacking information about outcomes of interest. Two authors (JHD and BJ) independently review the titles and abstracts of all identified studies for eligibility, excluding obviously ineligible ones. The eligibility of those remaining studies for final inclusion was further determined by examining the full text.

### Literature selection

All relevant studies were imported into Endnote X9, and then duplicate literature was excluded. Next, two researchers (JHD and BJ) independently excluded studies by reading titles and abstracts. At last, the irrelevant studies were removed that did not satisfy the PICOS. A senior reviewer (YTY) is consulted in cases of disagreement.

### Data abstraction

Two authors (JHD and BJ) independently performed data extraction: author, year of publication, journal of included studies, total number of patients, number of patients in group amiodarone (GA) and group control (GC), gender, age, surgical procedure, and data regarding outcomes of interest. Disagreements were resolved by discussion among all authors during the process of data abstraction.

### Quality assessment

The risk of bias was evaluated for RCTs by two authors (JHD and BJ), which consisted of the following items: sequence generation, allocation concealment, blinding of participants, blinding of outcome assessors, incomplete outcome data, reporting bias, and other bias, based on the Cochrane Handbook for Systematic Reviews of Interventions version [16]. Additionally, the modified *Jadad* score [17] was used independently by two authors (JHD and BJ) to evaluate the methodologic quality of each included trial.

### Data analysis and statistical methods

All data were analyzed by utilizing RevMan 5.3 (Cochrane Collaboration, Oxford, UK). Pooled risk ratio (RR) and 95% confidence interval (CI) were estimated for dichotomous data and the weighted mean difference (WMD) and 95% CI for continuous data, respectively. Each outcome was tested for heterogeneity, and a random effects model or fixed effects model was used in the presence or absence of significant heterogeneity (Q-statistical test *P* < 0.05). Sensitivity analyses were done by examining the influence of the statistical model on estimated treatment effects, and analyses that adopted the fixed effects model were repeated again using random effects model and vice versa. In addition, sensitivity analysis was also performed to evaluate the influence of individual studies on the overall effects. Subgroup analyses were performed to evaluate the possible effects of patient characteristics and control agents on the outcomes, if necessary. Publication bias was explored through visual inspection of funnel plots of the outcomes. All P values were 2-sided, and statistical significance was defined as* P* < 0.05.

### Subgroup analysis and investigation of heterogeneity

The trials were divided into two subgroups according to intra-, and post-operative AF and any arrhythmia. Meanwhile, the trials were divided into two subgroups according to pre-, and post-dose HR, MAP, and PH.

## Results

### Search results

**Fig. 1:**
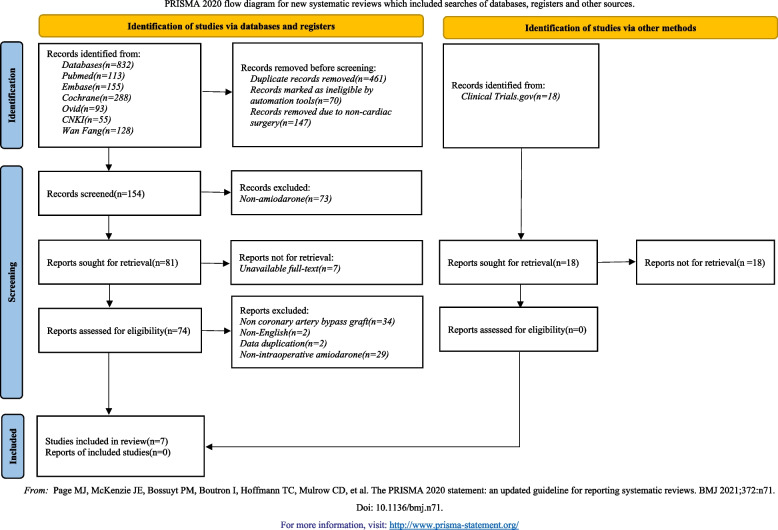
Flow chart

832 relevant studies were collected from databases (Pubmed, Embase, Cochrane Library, Ovid, CNKI, and Wan Fang) based on the search strategies. Additional records were identified through other sources (reference lists, *n* = 18). We used Endnote Software (Version X9, Thompson Reuters, CA) to remove 461 duplicate studies. According to the title and abstract, 315 relevant studies were excluded, and then 67 studies were removed by reading the full text. Finally, seven studies [18–24] were included in this meta-analysis in accordance with the inclusion criteria. The PRISMA flow diagram is listed (Fig. 1).

### Included trial characteristics

The basic information of the seven studies, including 608 patients, was shown (Table 1). All of them evaluated the efficacy of amiodarone, and lidocaine, or placebo for on-pump CABG. All seven RCTs included in the literature were published between 2003 and 2018. All literature were randomized double-blind studies, all of which included adults. All patients included were on pump CABG alone, and cardioplegia was used during surgery. Four studies reported the dose of intraoperative intravenous injection of amiodarone (150 mg), and another three studies reported the dose of amiodarone (300 mg). Three studies were conducted in Iran; two studies were conducted in the USA; and two studies were conducted in Turkey. Three pieces of literature evaluated AF, and four pieces evaluated VF. Due to CABG patients being monitored in the ICU for 24 h after surgery, ICU nurses recorded any postoperative arrhythmias within 24 h. Patients were monitored for ECG in real-time.

**Table 1:** Characteristics of included trials

**Trials**	**Sample size**	**Surgery**	**Male, n (%)**		**Weight (kg)**		**Height (cm)**		**Protocols(n)**		**Outcomes**
			**GA**	**GC**	**GA**	**GC**	**GA**	**GC**	**GA **	**GC **	
Kashani 2018[18]	74	OPCAB （Adults）	25(69)	22(57)	62±12	61±12	163±10	161±11	300 mg *iv*, before ACCR(n=37)	Lidocaine 100 mg *iv*(n=37)	①②③④⑥⑨⑩
Esmail 2015[19]	124	OPCAB （Adults）	39(63)	34(55)	NM	NM	NM	NM	300 mg* iv*, before AI(n=62)	Saline(n=62)	①③⑥
Yilmaz 2014[20]	86	OPCAB （Adults）	20(81)	①22(76) ②24(80)	78±16	①78±13 ②77±16	169±5	①169±8 ②169±8	300 mg *iv, *before ACCR(n=27)	①Lidocaine 1.5mg/kg *iv*(n=29) ②Saline(n=30)	②④⑤⑦⑨⑩
Alireza 2013[21]	150	OPCAB （Adults）	39(78)	①40(80) ②43(86)	71±11	①75±16 ②71±12	162±24	①164±16 ②166±9	150 mg *iv*, before ACCR(n=50)	①Lidocaine 100 mg iv(n=50) ②Saline(n=50)	②④⑨
Rahman 2009[22]	24	OPCAB （Adults）	9(60)	120(67)	NM	NM	NM	NM	150 mg *iv*, after AI(n=12)	Saline(n=12)	⑤⑥⑧
Ayoub 2009[23]	120	OPCAB （Adults）	36(90)	①37(93) ②36(90)	NM	NM	NM	NM	150 mg *iv*, before ACCR(n=40)	①Lidocaine 100 mg *iv* (n=40) ②Saline(n=40)	②④
Cheung 2003[24]	30	OPCAB （Adults）	13(87)	13(87)	85±17	89±12	NM	NM	150 mg *iv, *after SC(n=15)	Saline(n=15)	①⑥⑩

Reported outcomes: ① = Atrial fibrillation; ② = Ventricular fibrillation; ③ = Any arrhythmia; ④ = Defibrillation; ⑤ = Length of stay; ⑥ = Hemodynamic parameter (blood pressure and heart rate); ⑦ = Mortality; ⑧ = Plasma levels of pro- and anti-inflammatory biomakers; ⑨ = Arterial blood gas; ⑩ = Inotropic requirement

*ACCR* Aortic cross-clamping release, *AI* Anesthesia induction, *GA* Group amiodarone, *GC* Group control, *IV* Intravenous injection, *OPCAB* On-pump coronary artery bypass grafting, *SC* sternal closure, *NM* Not mentioned

### Risk of bias in included studies

**Fig. 2:**
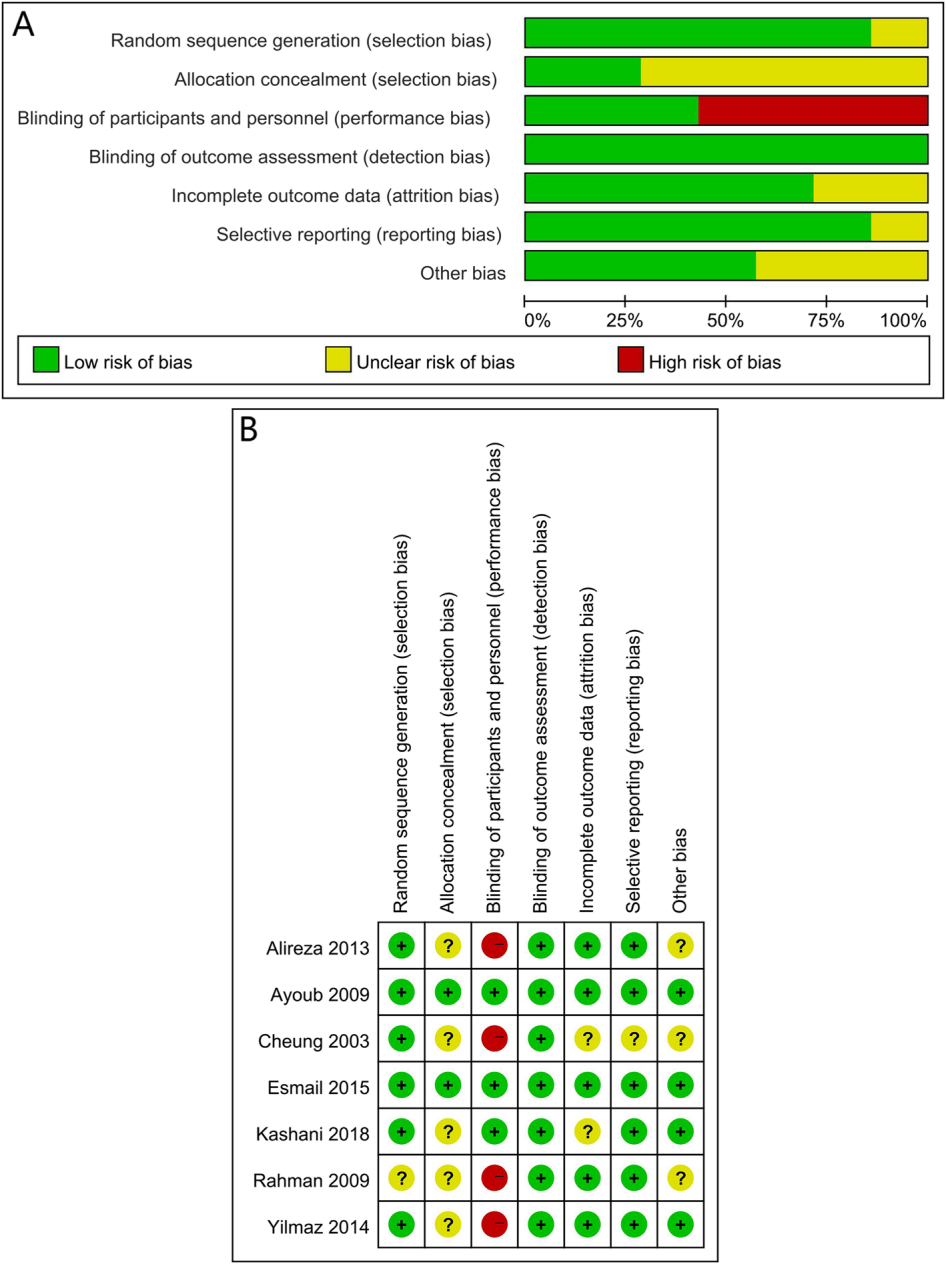
**A** Risk of bias graph for each included study. **B** Risk of bias summary for each included study

Details regarding the performance of the studies against each domain were presented in the risk of bias graph (Fig. 2A). Additionally, a visual summary of judgment about each methodological quality item for each included trial was shown (Fig. 2B). Of the seven RCTs, two studies did not report the random sequence generation and thus should be listed as unclear in terms of the risk of bias. Five studies did not report the allocation concealment and should be listed as having an unclear risk of bias. Because anesthesiologists find it difficult to lose sight unless they use carefully designed placebos to prevent them from knowing which medication to administer, if a blinding strategy is not specified, it should be deemed high-risk. Four studies did not report the blinding of participants and personnel and should be listed as having a high-risk of bias (Fig. 2B). Of the seven included trials, five trials [18–21, 23] had *Jadad* scores ≥ 4 and were considered high-quality RCTs, two trials [22, 24] had *Jadad* scores = 3 (Supplement Table 2).

### Effects on AF, VF, and any arrhythmia

**Fig. 3:**
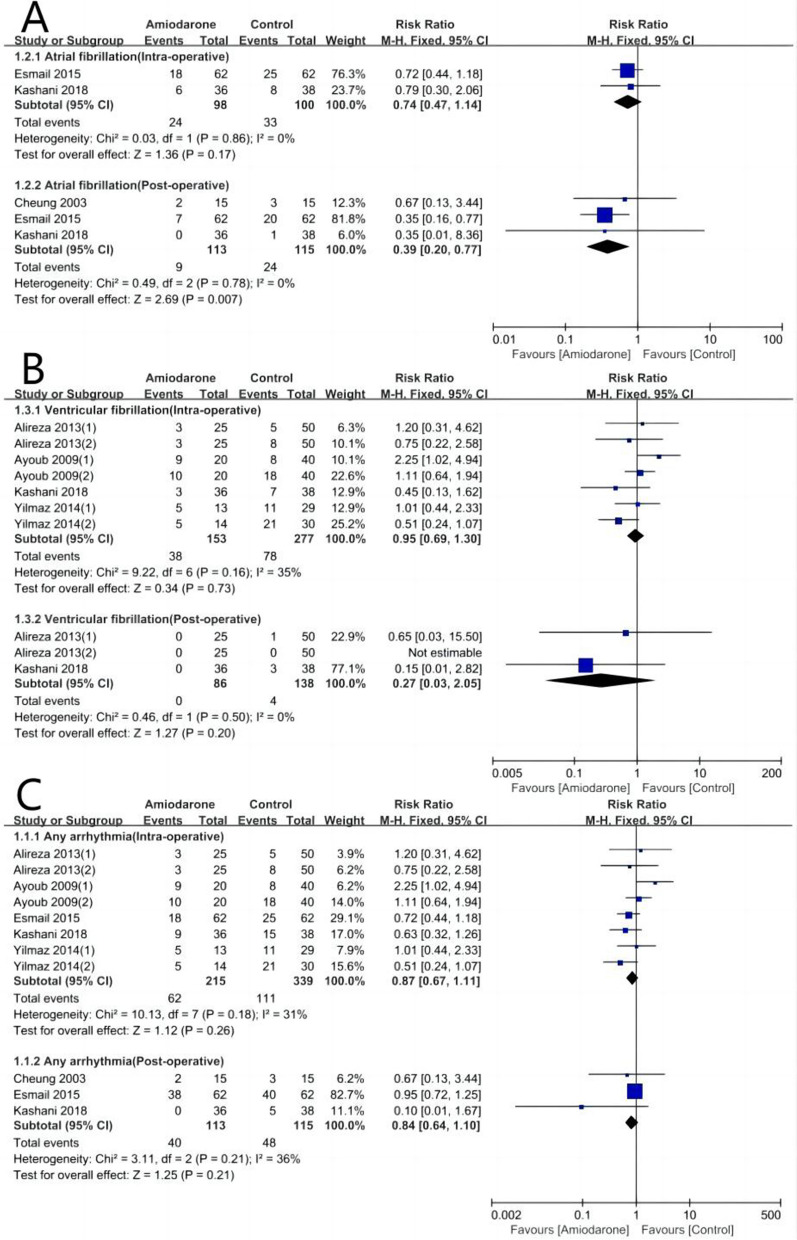
Forest plot comparing amiodarone and control for the incidences of atrial fibrillation (**A**), ventricular fibrillation (**B**), and any arrhythmia (**C**)

Three studies, including three treatments (amiodarone, lidocaine, and saline), contributed to the clinical outcome of POAF. Meta-analysis demonstrated that amiodarone can significantly reduce the incidence of POAF in patients undergoing CABG [RR, 0.39; 95% CI: 0.20, 0.77; *P* = 0.007] with heterogeneity [*I*^*2*^ = 0%, *P* = 0.78]. However, amiodarone achieved no statistically significant influence on the intraoperative AF [*n* = 2 trials; RR, 0.74; 95% CI: 0.47, 1.14; *P* = 0.17] with heterogeneity [*I*^*2*^ = 0%, *P* = 0.86] (Fig. 3A).

Four studies, including three treatments (amiodarone, lidocaine, and saline), contributed to the clinical outcome of VF. Meta-analysis demonstrated that amiodarone achieved no statistically significant influence on the VF (intraoperative: [RR, 0.95; 95% CI: 0.69, 1.30; *P* = 0.73] with heterogeneity [*I*^*2*^ = 35%, *P* = 0.16]); postoperative: [RR, 0.27; 95% CI: 0.03, 2.05; *P* = 0.20] with heterogeneity [*I*^*2*^ = 0%, *P* = 0.50]) (Fig. 3B).

Five studies, including three treatments (amiodarone, lidocaine, and saline), contributed to the clinical outcome of any arrhythmia. Meta-analysis demonstrated that amiodarone achieved no statistically significant influence on any arrhythmia (intraoperative: [RR, 0.87; 95% CI: 0.67, 1.11; *P* = 0.26] with heterogeneity [*I*^*2*^ = 31%, *P* = 0.18]; postoperative: [RR, 0.84; 95% CI: 0.64, 1.10; *P* = 0.21] with heterogeneity [*I*^*2*^ = 36%, *P* = 0.21]) (Fig. 3C).

### Defibrillation and inotropic support

Four studies, including three treatments (amiodarone, lidocaine, and saline), contributed to the clinical outcome of defibrillation after ACCR. Meta-analysis demonstrated that amiodarone achieved no statistically significant influence on defibrillation [RR, 0.82; 95% CI: 0.55, 1.20; *P* = 0.31] with heterogeneity [*I*^*2*^ = 46%, *P* = 0.09] (Supplement Fig. 1A).

Two studies, including three treatments (amiodarone, lidocaine, and saline), contributed to the clinical outcome of the highest energy used for defibrillation. Meta-analysis demonstrated that amiodarone achieved no statistically significant influence on the highest energy used for defibrillation [WMD = -5.53; 95% CI: -12.39, 1.33; *P* = 0.11] with heterogeneity [*I*^*2*^ = 83%, *P* = 0.02] (Supplement Fig. 1B).

Three studies, including three treatments (amiodarone, lidocaine, and saline), contributed to the clinical outcome of inotropic requirement after ACCR. Meta-analysis demonstrated that AM achieved no statistically significant influence on the inotropic requirement after ACCR [RR, 0.99; 95% CI: 0.69, 1.41; *P* = 0.94] with heterogeneity [*I*^*2*^ = 0%, *P* = 0.50] (Supplement Fig. 1C).

### HR, MAP, and PH

Four trials (4 comparisons, 252 patients), three trials (3 comparisons, 222 patients), and three trials (4 comparisons, 310 patients) reported pre- and post-dose of HR, MAP, and PH, respectively (Table 1). Meta-analysis demonstrated that GA had comparable HR (pre-dose) [WMD = -1.99; 95% CI: -6.71, 2.72; *P* = 0.41] with heterogeneity [*I*^*2*^ = 62%, *P* = 0.05]; HR (post-dose) [WMD = -11.35; 95% CI: -26.95, 4.25; *P* = 0.15] with heterogeneity [*I*^*2*^ = 97%, *P* < 0.00001]; MAP (pre-dose) [WMD = -0.04; 95% CI: -3.79, 3.71; *P* = 0.98] with heterogeneity [*I*^*2*^ = 46%, *P* = 0.16]; MAP (post-dose) [WMD = -2.37; 95% CI: -9.87, 5.12; *P* = 0.53] with heterogeneity [*I*^*2*^ = 78%, *P* = 0.01]; PH (pre-dose) [WMD = -0.00; 95% CI: -0.02, 0.01; *P* = 0.68] with heterogeneity [*I*^*2*^ = 0%, *P* = 0.46]; PH (post-dose) [WMD = -0.01; 95% CI: -0.02, 0.01; *P* = 0.55] with heterogeneity [*I*^*2*^ = 59%, *P* = 0.09] to GC (Supplement Fig. 2).

### Postoperative recovery

One trial (1 comparison, 24 patients), two trials (3 comparisons, 110 patients), and two trials (3 comparisons, 110 patients) reported MVD, LOS in the ICU, and LOS in the hospital, respectively (Table 1). Meta-analysis demonstrated that GA had comparable MVD [WMD = 0.49; 95% CI: -2.70, 3.68; *P* = 0.76]; LOS in the ICU [WMD = -0.06; 95% CI: -0.02, 0.07; *P* = 0.37] with heterogeneity [*I*^*2*^ = 5%, *P* = 0.35]; LOS in the hospital [WMD = -0.03; 95% CI: -0.43, 0.37; *P* = 0.90] with heterogeneity [*I*^*2*^ = 0%, *P* = 0.87] to GC (Supplement Fig. 3).

### Sensitivity analyses and publication bias

Sensitivity analyses showed that treatment effects on all the outcomes were not affected by the choice of statistical models (Supplement Tables 3, 4). Sensitivity tests were also performed by the exclusion of some studies to analyze the influence of the overall treatment effect on high heterogeneity outcomes (Supplement Table 5), but no contradictory results were found. Meanwhile, we found that there may be a small sample effect or little publication bias (Fig. 2). No significant publication bias was detected by the funnel plot examination for AF and VF (Supplement Fig. 4).

## Discussion

To our best knowledge, this is the first meta-analysis dedicated to evaluating the efficacy and safety of intraoperative intravenous injections of amiodarone for on-pump CABG patients. Amiodarone administration can reduce the incidence of POAF in patients undergoing CABG. However, amiodarone administration can't significantly influence the AF (intraoperative), VF, or any arrhythmia (intraoperative and postoperative).

AF is one of the most common postoperative complications following cardiac surgery. This in turn translates into longer hospitalization, an increased cost of hospitalization, as well as an association with thromboembolic events and mortality [25–27]. In the comparison of GA and group lidocaine [18]/saline [19], the prevalence of AF is 16.7% vs. 21.1% [18] and 29% vs. 40.3% [19] (*P* = 0.17*)* intraoperatively, respectively. Our meta-analysis results are not consistent with this study showing that AF was observed in 6 patients (14.3%) in the GA and 15 patients (37.5%) in the GC (*P* = 0.035) [28]. This may be due to the relatively small number of patients included; although the incidence of intraoperative AF in the GA was lower than that in the GC, the statistical difference was not achieved.

POAF after CABG was common in modern research. A recently published study (2022) showed that the incidence of POAF in CABG was 20.9% [29]. Patients with POAF after CABG had three times the incidence of long-term AF compared with both non-POAF patients and matched controls [30]. A total of three studies [18, 19, 24], including three treatments (amiodarone, lidocaine, and saline), contributed to the clinical outcome of POAF. Meta-analysis demonstrated that amiodarone administration can significantly reduce the incidence of POAF in patients undergoing CABG; the prevalence of POAF is 0%, 11.3%, and 13.3% vs. 2.6%, 32.3%, and 20%, respectively. Our meta-analysis results are consistent with these studies, showing that new-onset POAF occurs in 20%-40% of patients [31–33] following CABG. Meanwhile, a recently published meta-analysis demonstrated that combination prophylaxis with amiodarone and beta-blockers significantly lowered the risk of POAF incidence in comparison to beta-blockers alone [34]. However, the underlying pathophysiological mechanisms of POAF are complex and involve the interaction between various triggers such as acute inflammation, cardiac sympathetic activation, and oxidative stress from one side and multiple pre-existing cardiac conditions such as structural heart abnormalities, ion channel disorders, and atrial interstitial alterations from the other side [35]. Similarly, it was demonstrated that the oxidative stress after cardiac tissue reperfusion in patients undergoing CABG can increase nicotinamide adenine dinucleotide phosphate (NADPH) oxidase activity in the right atrial appendage tissue, which was shown to be the most important independent predictor of developing POAF [36]. Meta-analysis demonstrated amiodarone effective in lowering POAF but not intra-operative AF rate. This is an interesting phenomenon that may exist for the following reasons: 1. In clinical studies, besides intervention measures, there are many influencing factors, such as the protective effect of cardioplegia techniques used in extracorporeal circulation on myocardium, which directly affects the incidence of AF after cardiac resurrection; 2. The administration time and dosage of amiodarone in the included studies are not completely consistent, which may affect its preventive effect on AF; 3. The number of studies and patients included in this study is limited, and more high-quality RCTs are needed to further elucidate this situation. In addition, for asymptomatic patients within POAF, rate control is sufficient to manage POAF in the included studies, and routine rhythm control is not needed. For patients with complications such as hemodynamic instability, rhythm control should be retained, and medication or electroconversion can be chosen. Anticoagulant therapy can be performed on postoperative POAF patients. The management of the above POAF is similar to the conventional management methods provided by the latest systematic review [37].

As depicted in Table 1, four trials [18, 20, 21, 23] (7 comparisons, 430 patients) and six trials [18–21, 23, 24] (8 comparisons, 584 patients) reported VF and any arrhythmia, respectively. The arrhythmias include: AF, VF, VT, PVC, and SB. Compared with the GC, the overall incidence of VF and any arrhythmia in the experimental group was 24.8% vs. 28.2% (*P* = 0.73) and 28.8% vs. 32.7% *(P* = 0.26), respectively. Our meta-analysis demonstrated that AM cannot reduce the incidence of intraoperative VF or any arrhythmia. The incidence of VF after ACC release is reported to vary with the experience of the surgeon and the category of surgical procedures, ranging from 45 to 90% in patients undergoing CABG [38, 39]. Moreover, another study indicated that administration of lidocaine with a perfusion pump before ACCR reduced VF incidence from 11 to 70% [6]. As for amiodarone, subsequent studies demonstrated that this medication may achieve comparable [40] or even greater [41] preventative effects against VF in patients who are at risk for the development of VF and pulseless VT during cardiac surgery. In comparison with the above literature, our meta-analysis demonstrated that the overall incidence of VF in the experimental group is relatively low. On the other hand, consistent with our META analysis results, sustained VT and VF occur rarely after CABG [42–46].

A study included indicates that the need for defibrillation was significantly higher in group lidocaine; additionally, the amount of energy (joules) needed for defibrillation was not significantly different between the two groups. The number of patients who needed inotropic requirements after the ACCR period was higher in the GA; however, it did not reach a significant level [19]. The study by Yilmaz et al. [20] demonstrated that when VF occurred, the percentage of patients requiring electrical defibrillation was significantly higher in both group lidocaine and GC when compared with GA (*P* = 0.023). however, another study by Ayoub et al. [23] demonstrated that when VF occurred, the percentage of patients requiring defibrillation counter shocks was significantly higher in both the amiodarone (58%) and control (61%) groups as compared with the lidocaine group (13%), with no difference between the amiodarone and the GC, despite a significant decrease in the defibrillation counter shock energy requirements in the GA.

Interestingly, several studies [18–22, 24] have suggested that there was no significant difference between the study and GC in terms of HR, MAP, and PH. The reason for consideration is that there are too few included literature and there is a high degree of heterogeneity.

POAF has been shown to increase the length of hospital stays, leading to higher health care costs [47]. However this two included pieces of literature indicate that amiodarone administration did not induce any difference in the MVD, LOS in the ICU, or LOS in the hospital [20, 22]. There is also a need for a large number of RCTs to be verified.

## Limitations

Our systematic review and meta-analysis have some limitations: Firstly, only 7 studies were included in our meta-analysis; the sample size was relatively small; if more studies had been contained, the statistical efficacy of our analysis would increase. Secondly, only English publications were included in our meta-analysis; thus, publication bias was unavoidable. Thirdly, all included studies lacked long-term follow-up. Long-term follow-up studies should be conducted in the future. Fourthly, the intervention strategies included in the studies were different, and there was heterogeneity. Finally, the optimal protocols and dosages for the administration of amiodarone and lidocaine during the perioperative period remain to be determined in future studies. Despite the above limitations, this is the most recent meta-analysis to evaluate the efficacy and safety of intraoperative intravenous injections of amiodarone for on-pump CABG patients. There is also a need for a large number of RCTs to be verified.

## Conclusions

The current study suggests that intraoperative administration of intravenous amiodarone may be safe and effective in preventing POAF in patients undergoing CABG. More well-designed clinical trials are needed to validate this result.

### Supplementary Information

**Additional file 1:** **Supplement Table 1. **Database search strategies.

**Additional file 2: Supplement Table 2. **Modified Jadad score of the included RCTs.

**Additional file 3: Supplement Figure 1. **Forest plot comparing amiodarone and control for defibrillation after ACCR (**A**), defibrillation energy (**B**), and inotropic requirement after ACCR (**C**).

**Additional file 4: Supplement Figure 2. **Forest plot comparing amiodarone and control for MAP (**A**), HR (**B**), and PH (**C**).

**Additional file 5: Supplement Figure 3. **Forest plot comparing amiodarone and control for MVD (**A**), LOS in the ICU (**B**), and LOS in the hospital (**C**).

**Additional file 6: Supplement Table 3. **Influence of a statistical model on estimated treatment effects of primary outcomes.

**Additional file 7: Supplement Table 4. **Sensitivity analyses of the influence of individual studies on the overall effects.

**Additional file 8: Supplement Table 5. **Sensitivity analyses of high heterogeneity outcomes.

**Additional file 9: Supplement Figure 4. **Funnel plot (atrial fibrillation,ventricular fibrillation, and any arrhythmia).

## Data Availability

The original contributions presented in the study are included in the article or supplementary material; further inquiries can be directed to the corresponding author.
