# Supplementary figures and images for: The efficacy and safety of intraoperative intravenous amiodarone in patients undergoing on-pump coronary artery bypass grafting surgery: a systemic review and PRISMA-compliant meta-analysis
Source: J Cardiothorac Surg. 2024 May 3;19:274. doi: 10.1186/s13019-024-02732-9 (PMC11067272; doi:10.1186/s13019-024-02732-9)

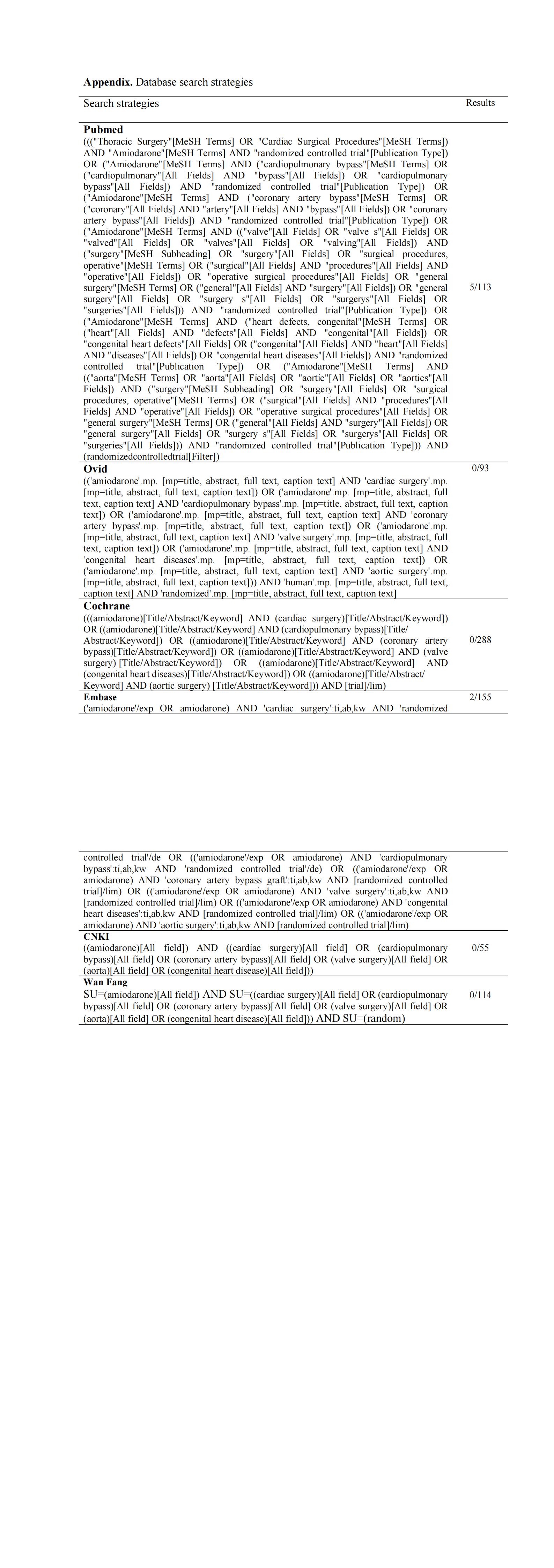

Supplement: Supplementary file 1 — Additional file 1: Supplement Table 1. Database search strategies. [file 13019_2024_2732_MOESM1_ESM.jpg]

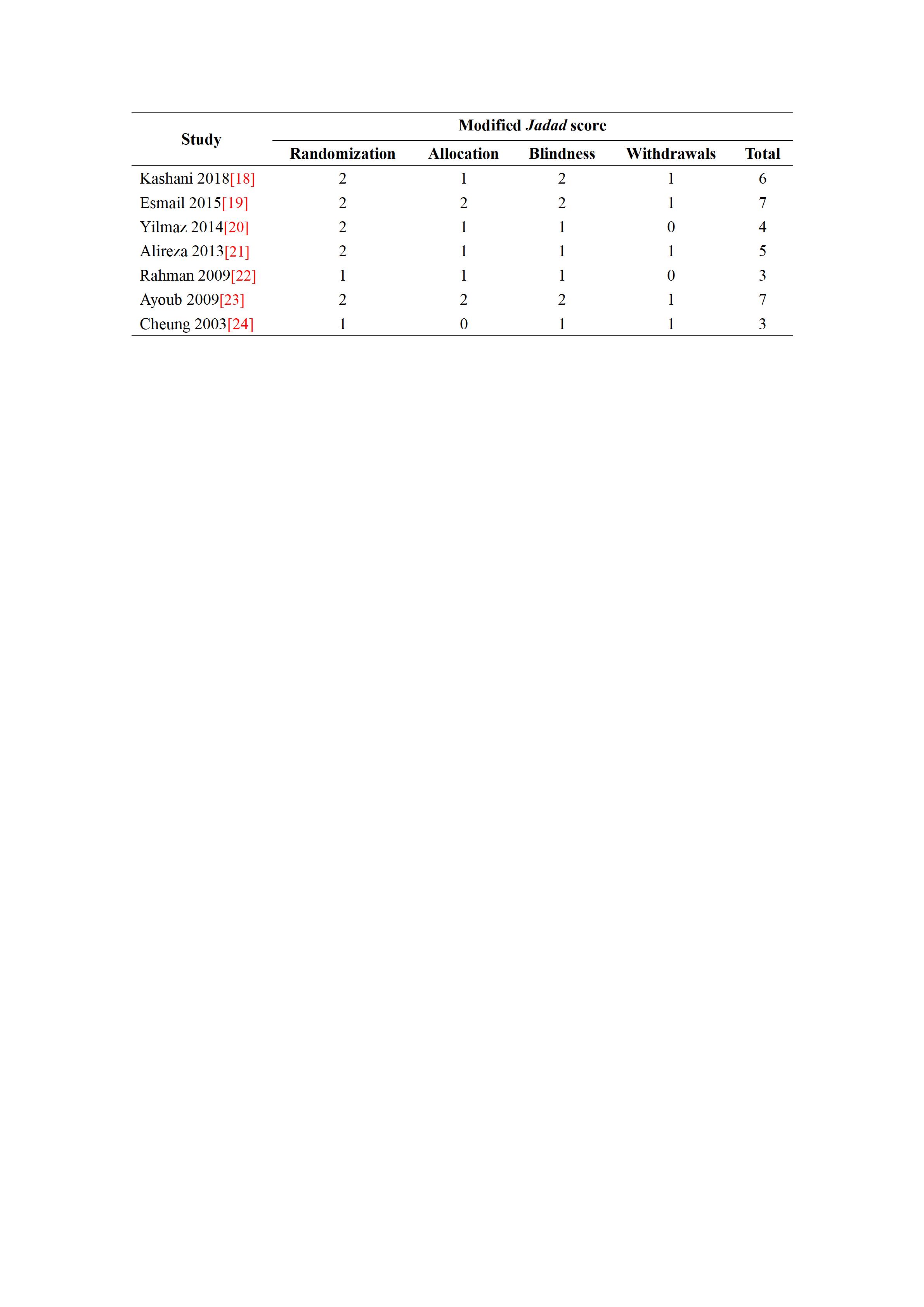

Supplement: Supplementary file 2 — Additional file 2: Supplement Table 2. Modified Jadad score of the included RCTs. [file 13019_2024_2732_MOESM2_ESM.jpg]

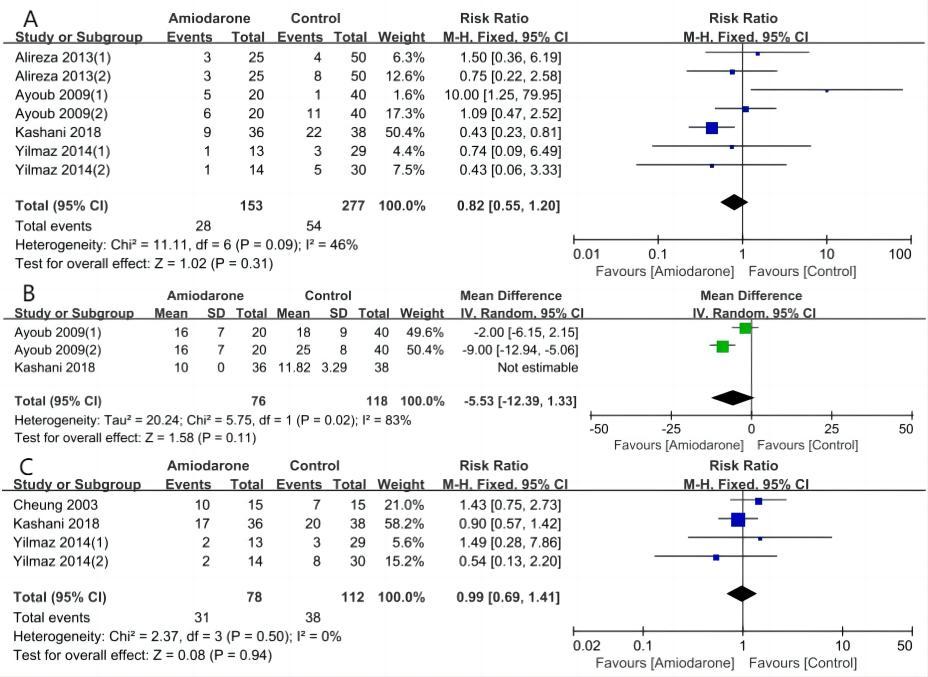

Supplement: Supplementary file 3 — Additional file 3: Supplement Figure 1. Forest plot comparing amiodarone and control for defibrillation after ACCR (A), defibrillation energy (B), and inotropic requirement after ACCR (C). [file 13019_2024_2732_MOESM3_ESM.jpg]

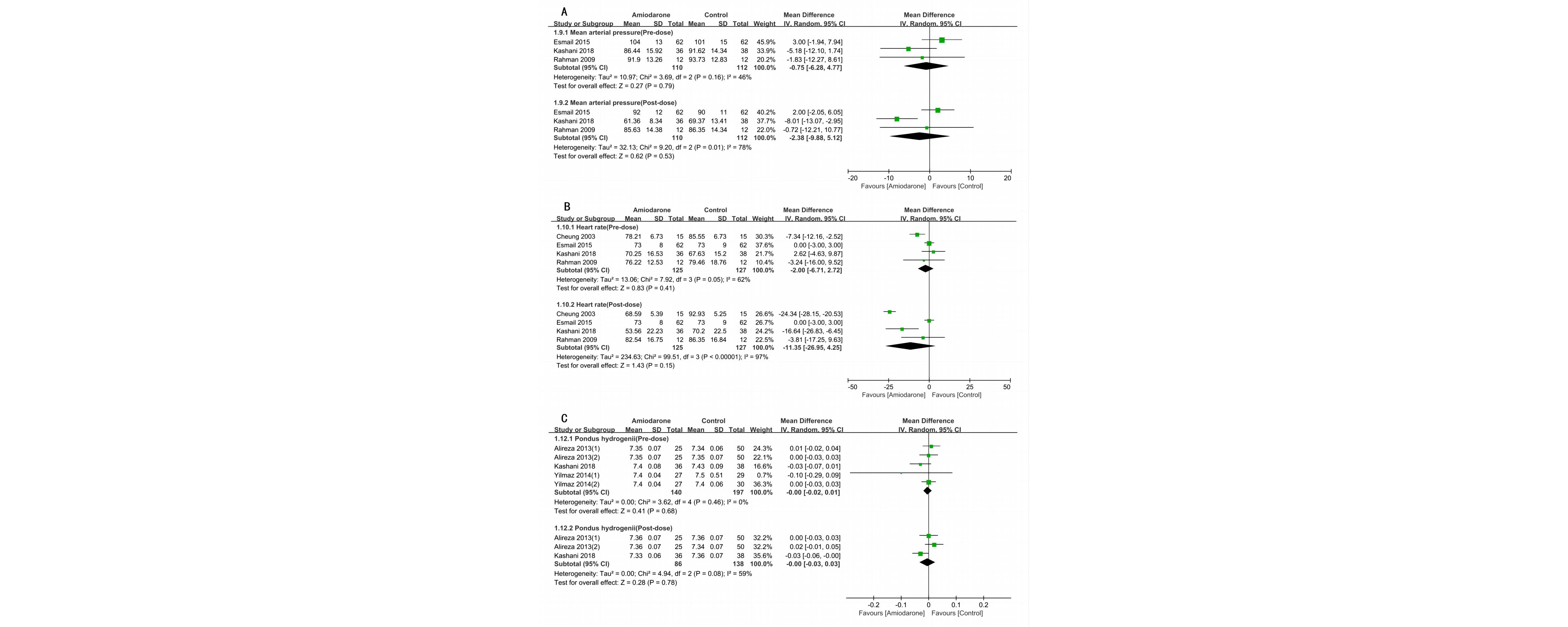

Supplement: Supplementary file 4 — Additional file 4: Supplement Figure 2. Forest plot comparing amiodarone and control for MAP (A), HR (B), and PH (C). [file 13019_2024_2732_MOESM4_ESM.jpg]

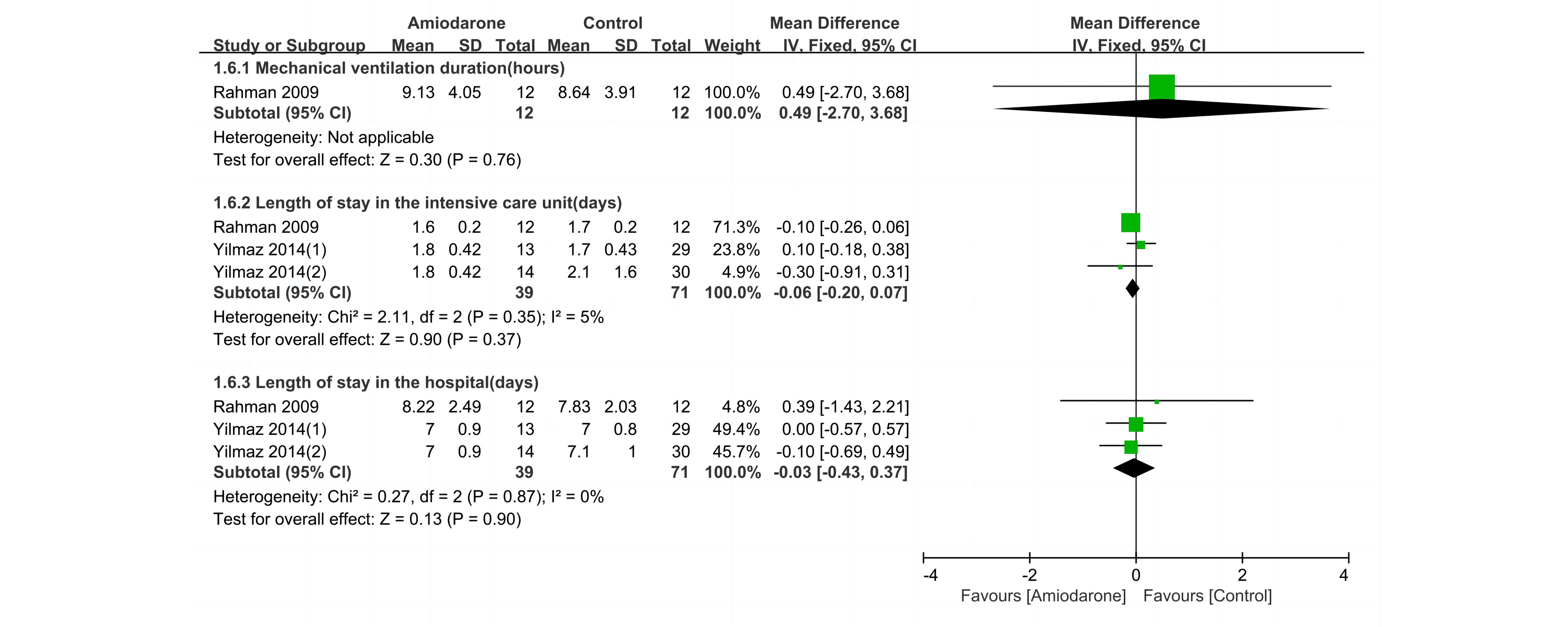

Supplement: Supplementary file 5 — Additional file 5: Supplement Figure 3. Forest plot comparing amiodarone and control for MVD (A), LOS in the ICU (B), and LOS in the hospital (C). [file 13019_2024_2732_MOESM5_ESM.jpg]

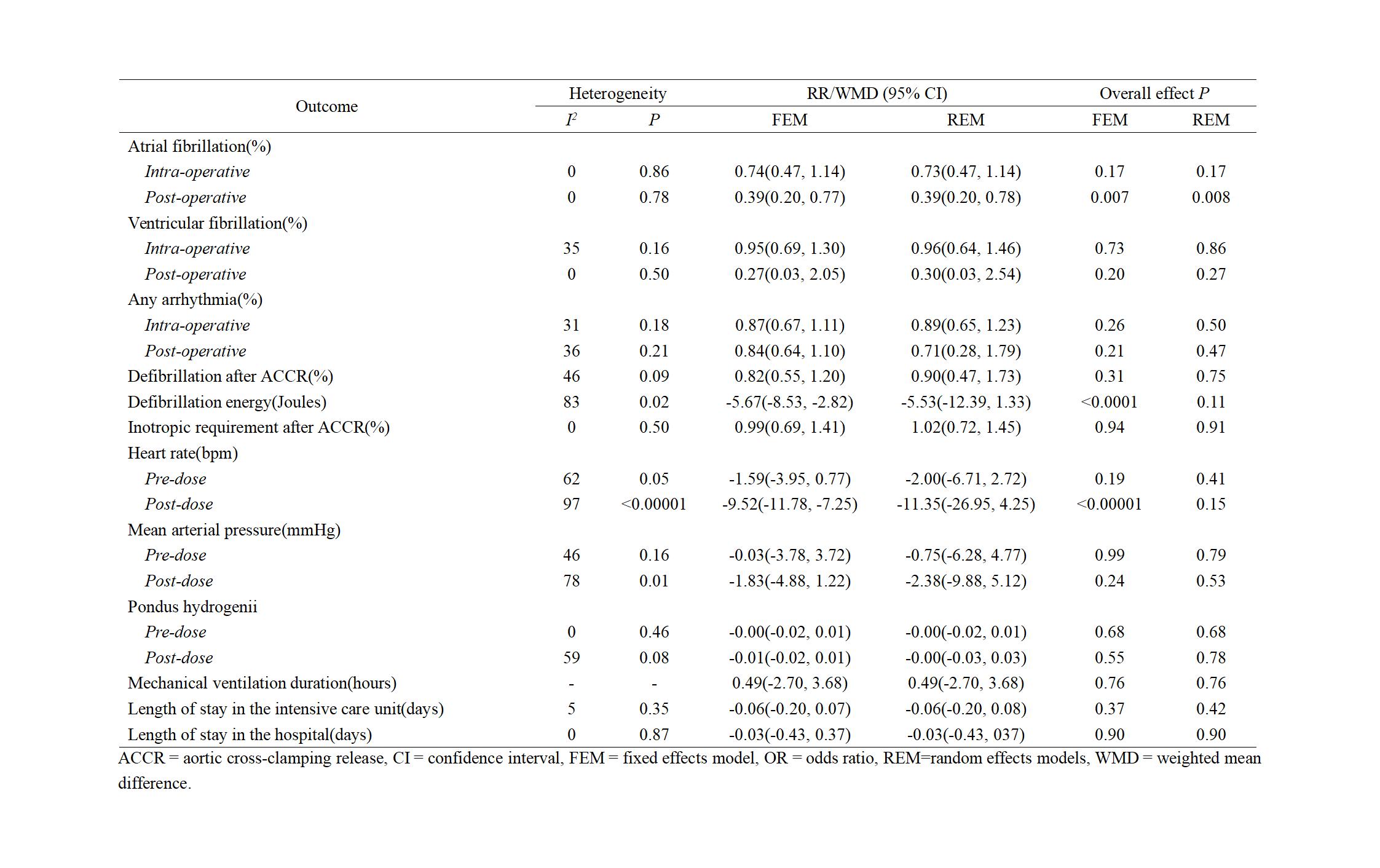

Supplement: Supplementary file 6 — Additional file 6: Supplement Table 3. Influence of a statistical model on estimated treatment effects of primary outcomes. [file 13019_2024_2732_MOESM6_ESM.jpg]

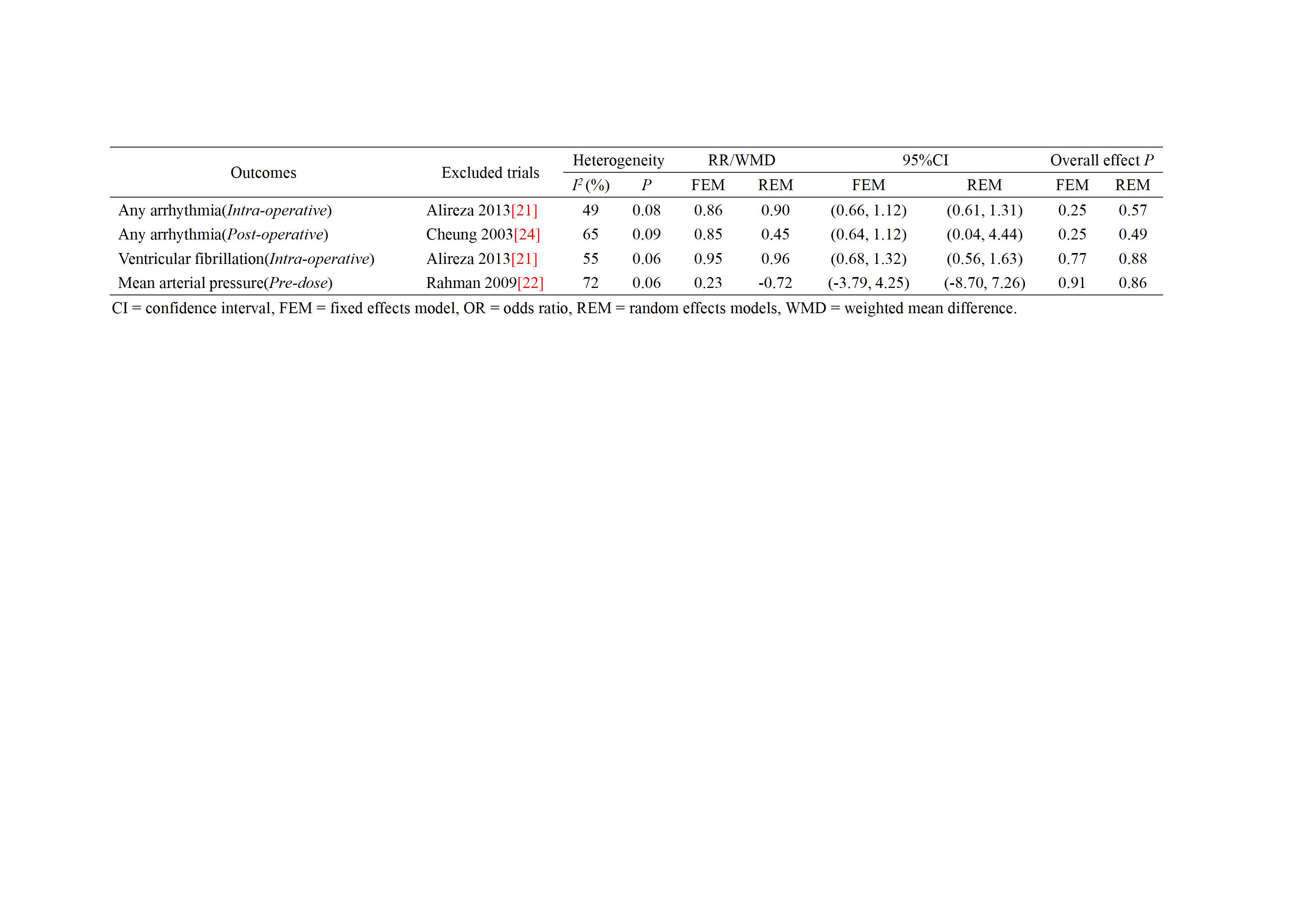

Supplement: Supplementary file 7 — Additional file 7: Supplement Table 4. Sensitivity analyses of the influence of individual studies on the overall effects. [file 13019_2024_2732_MOESM7_ESM.jpg]

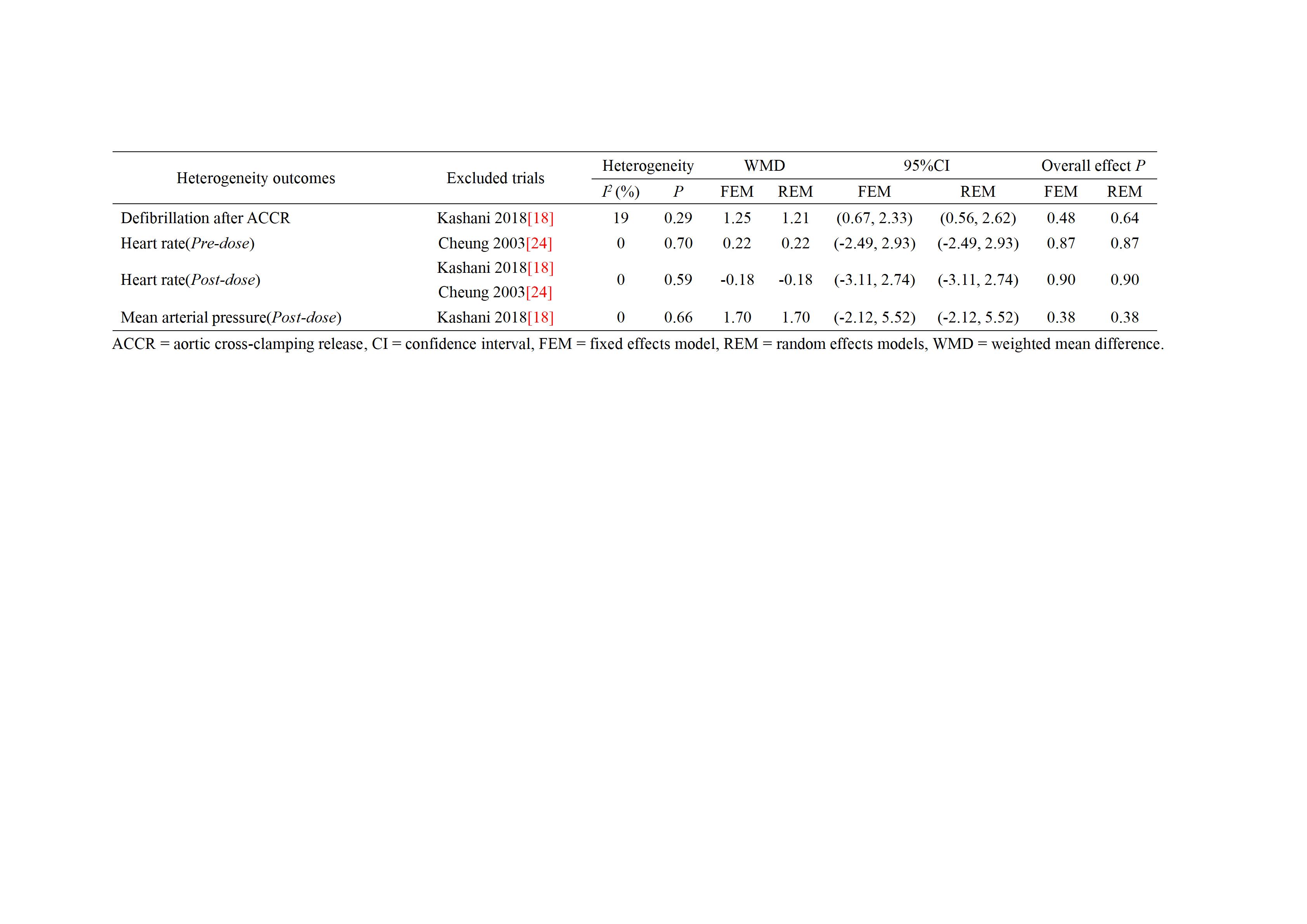

Supplement: Supplementary file 8 — Additional file 8: Supplement Table 5. Sensitivity analyses of high heterogeneity outcomes. [file 13019_2024_2732_MOESM8_ESM.jpg]

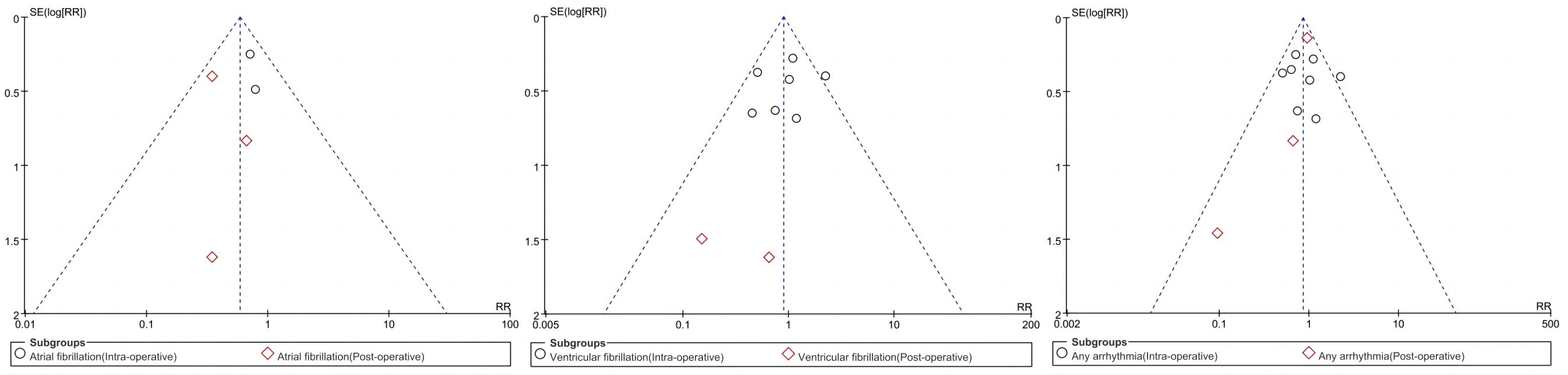

Supplement: Supplementary file 9 — Additional file 9: Supplement Figure 4. Funnel plot (atrial fibrillation,ventricular fibrillation, and any arrhythmia). [file 13019_2024_2732_MOESM9_ESM.jpg]
